# Non-doctoral factors influencing the surgical choice of Chinese patients with breast cancer who were eligible for breast-conserving surgery

**DOI:** 10.1186/s12957-019-1723-4

**Published:** 2019-11-11

**Authors:** Rui Chen, Sainan You, Zinan Yin, Qiannan Zhu, Chaojun Jiang, Shuo Li, Yan Li, Xiaoming Zha, Jue Wang

**Affiliations:** 1 Breast Disease Department, the First Affiliated Hospital of Nanjing Medical University, Nanjing, China; 2 Endocrine Department, Peking Union Medical College Hospital, Beijing, China

**Keywords:** Surgical choice, Breast-conserving surgery, Mastectomy, Breast cancer

## Abstract

**Background:**

The rate of breast-conserving surgery (BCS) is low in China. Many patients choose mastectomy even when informed that there is no difference in the overall survival rate compared with that of BCS plus radiotherapy. This study aimed to investigate the factors that influenced the surgical choice in patients eligible for BCS.

**Methods:**

Female patients with breast carcinoma were enrolled in a single center from March 2016 to January 2017. They made their own decision regarding the surgical approach. Univariate analysis was employed to determine the factors associated with the different breast surgical approaches. Significant factors (defined as *P* < 0.05) were then incorporated into multivariate logistic regression models to determine the factors that independently influenced patients’ decision.

**Results:**

Of the 271 patients included, 149 were eligible for BCS; 65 chose BCS and 84 chose mastectomy. On the basis of univariate analysis, patients with younger age, higher income and education, shorter admission to surgery interval, and shorter confirmed diagnosis to surgery interval were more likely to choose BCS than mastectomy (*P* < 0.05). Meanwhile, patients who resided in rural regions, did not have general medicare insurance, and were diagnosed with breast cancer preoperatively were more inclined to choose mastectomy than BCS (*P* < 0.05). The multivariate model revealed three independent influencing factors: age at diagnosis (*P* = 0.009), insurance status (*P* = 0.035), and confirmed diagnosis to surgery interval (*P* = 0.037). In addition, patients receiving neoadjuvant chemotherapy (NCT) were more inclined to choose mastectomy.

**Conclusion:**

Surgical choice of patients eligible for BCS was affected by several factors, and age at diagnosis, confirmed diagnosis to surgery interval, and insurance status were independent factors.

## Introduction

Breast cancer is one of the most common malignant tumors in women. Despite modern advances in medication, surgery still plays a vital role in breast cancer treatment, with common alternatives such as mastectomy and breast-conserving surgery (BCS). BCS followed by radiotherapy not only has been shown to have an equivalent disease-free survival rate and overall survival rate to mastectomy [1, 2] but also has the advantages of improving cosmetic outcome and reducing surgical complications [3, 4]. For these reasons, the US National Institutes for Health has recommended BCS for early breast cancer [5, 6], and more than 60% of patients with early-stage breast cancer undergo BCS in the USA [7]. In China, BCS is performed far less often [8], with a nationwide rate of only 11.2% [9].

A large proportion of Chinese women still choose mastectomy when diagnosed with breast cancer [8, 9]. Several demographic and socioeconomic factors have been proposed to explain this, including age, insurance status, level of education, marital status, and type of hospital [10–13]. Surgeons still play a leading role in the choice of surgical approach for patients in most regions of China, and many doctors recommend their patients to choose mastectomy even when they are suitable for BCS [9]. The reasons for this are complex. Some surgeons still consider the traditional opinion that BCS is not as safe as mastectomy in terms of recurrence rate in some remote areas [9]. In addition, the availability of BCS can be limited by local conditions. For example, some hospitals, particularly in rural regions, do not have facilities for postoperative radiotherapy. In many city and provincial hospitals, rapid pathological examination is routinely performed during surgery to determine the surgical margin. This rapid evaluation is not available in small hospitals, such as those at county level; hence, mastectomy is often recommended.

The aim of the present study was to analyze the factors that influence the surgical choices of breast cancer patients in China who were eligible for BCS. Our hospital has a specialized surgical team for breast cancer treatment and the technologies needed for postoperative radiotherapy, and intraoperative pathological examinations for BCS are routinely performed. Because these potential medical biases did not need to be considered, this study focused mainly on demographic and socioeconomic factors that influenced the surgical choices. We address this problem by examining the decision-making process prospectively and allowing the patients to make their own decisions.

## Materials and methods

Female patients with breast cancer who underwent breast surgery between March 2016 and January 2017 were included in this single-center study, and this pilot study was performed by one of the medical groups of our center. All the members of this medical group have vast experience and are aware about the latest concepts for breast cancer treatment, and the lead surgeon has at least 20 years of experience in managing breast disease. Each patient underwent a preoperative clinical evaluation by the same medical group to determine whether they were suitable for BCS. The eligibility criteria for BCS in Chinese Anti-Cancer Association, Committee of Breast Cancer Society (CACA-CBCS, 2017 version) were as follows: stage I/II cancer; tumors with a maximum diameter of < 3 cm, for which this approach is particularly suitable; and appropriate tumor to breast volume ratio. Moreover, patients with stage III cancer (except inflammatory breast cancer) who underwent preoperative neoadjuvant treatment following which the disease has reached BCS standard may be considered. Surgeons did not recommend BCS if there were absolute or relative contraindications. The patients eligible for BCS were given detailed information about the advantages or disadvantages of the two surgical methods, including that there was no difference in overall survival rate between the two approaches; they then made their own decision about the method to be used. Before surgery, each patient signed an informed consent form after careful consideration.

**Fig. 1:**
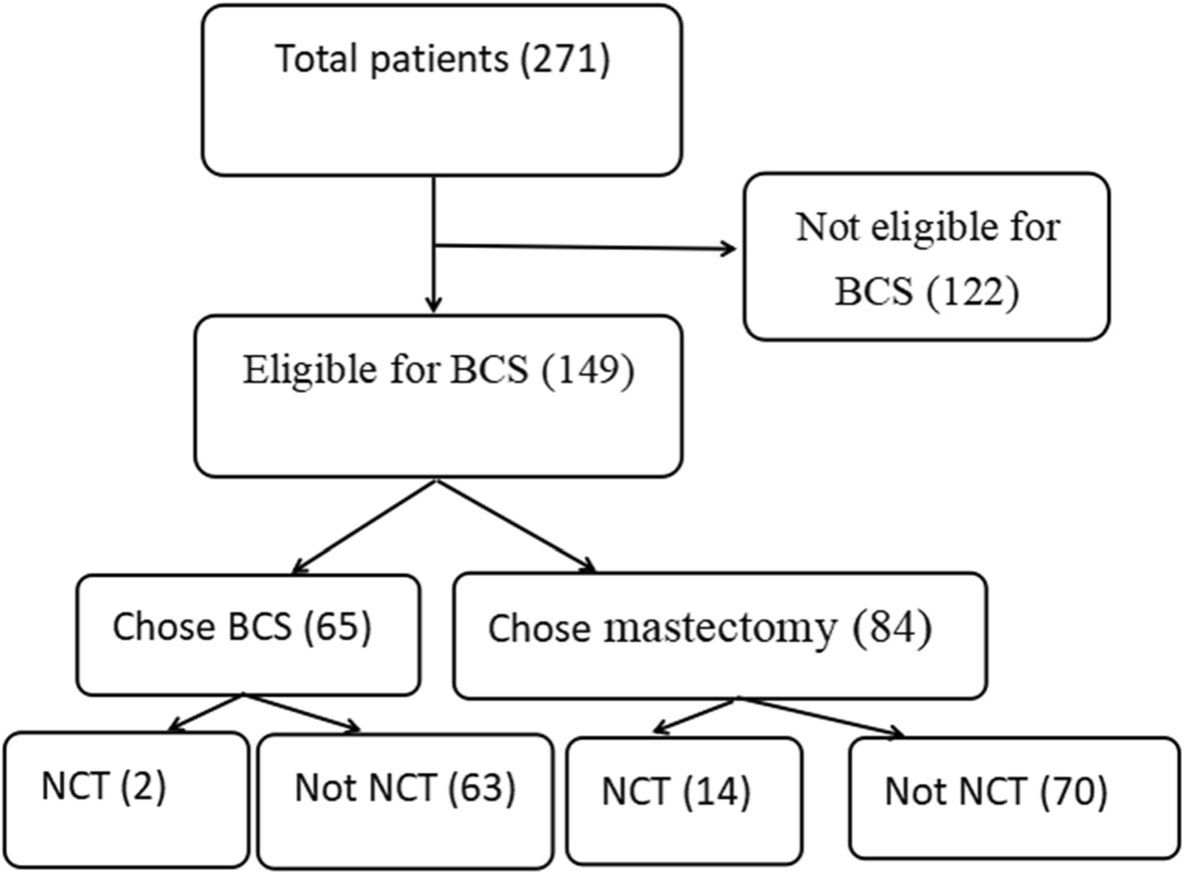
Flow diagram of the breakdown of patient cohort

The analysis in this study was based on the preoperative characteristics of the patients and their decision-making processes after they were fully informed about the two surgical methods. Age, menstrual status, residence, insurance status, income, level of education, preoperative pathology, and other factors were included in the analysis. A subanalysis included only the patients who underwent NCT. In addition, the patients who chose mastectomy were asked to complete a simple questionnaire about the reason for their choice. A flow diagram was used to illustrate the abovementioned content (Fig. 1).

Patient characteristics and related factors were summarized using basic descriptive statistics, including means, medians, and standard deviations. The area under the curve was used to calculate the cutoff value of confirmed diagnosis to surgery interval and admission to surgery interval (patients receiving NCT were excluded). Based on the calculated results, we approximately defined them as 3 days, and this did not affect the final result. Pearson’s chi-squared and *t* tests were used for the univariate analysis of variables potentially associated with choosing BCS versus mastectomy. The factors that were statistically significant (defined by *P* < 0.05, two-tailed) in the univariate analysis were then incorporated into binary logistic regression models to determine the factors that independently influenced the patients’ choice between BCS and mastectomy, calculating odds ratios (ORs) and 95% confidence intervals (CIs) for these factors. Patients who chose BCS and mastectomy were encoded as 0 and 1, respectively, and other categorical variables were also encoded (0, 1). The statistical analysis was performed using SPSS version 20.0 (IBM Corp.).

## Results

A total of 271 patients were included in the study. Of these, 149 patients were determined to be eligible for BCS; the other 122 patients had absolute or relative contraindications to BCS. Among the 149 eligible patients, 65 chose BCS and 84 chose mastectomy; thus, 24% of the 271 patients with breast cancer received BCS. Table 1 summarizes the results of the univariate analysis. The patients who chose BCS were younger than those who chose mastectomy (48.54 ± 9.52 vs 55.50 ± 14.03 years; *P* = 0.0008), but there was no significant difference between the groups in menstrual status, and no differences in tumor characteristics, including tumor size (17.32 ± 6.31 vs 18.10 ± 6.68 mm), node state, and pathological type before surgery (invasive carcinoma, carcinoma in situ, or other). BCS was chosen significantly more often by patients who resided in urban areas, those with general medicare insurance, those with a family income of > $1500 per year, and those educated to a bachelor’s degree or above. Whether or not the patient resided in Nanjing was not a significant factor.

**Table 1:** Univariate analysis of factors associated with the choice of BCS or mastectomy for patients eligible for BCS

Patient characteristics	BCS (*n* = 65)	Mastectomy (*n* = 84)	*P* value
Age, years			
Mean ± SD	48.54 ± 9.52	55.50 ± 14.03	0.0008*
Median (range)	47 (25–71)	55 (28–85)	
Menstrual status, years, no. (%)			
Premenopausal	38 (58.5)	36 (42.9)	0.07
Postmenopausal	27 (41.5)	48 (57.1)	
Place of residence, no. (%)			
Urban	61 (93.8)	52 (61.9)	< 0.001*
Rural	4 (6.2)	32 (38.1)	
Within Nanjing region	37 (56.9)	53 (63.1)	0.5007
Outside Nanjing	28 (43.1)	31 (36.9)	
Insurance status, no. (%)			
General medicare insurance	61 (93.8)	43 (51.2)	< 0.001*
No or low-level medicare insurance	4 (6.2)	41 (48.8)	
Household income, $, no. (%)			
> 1500 per year	41 (63.1)	18 (21.4)	< 0.001*
≤ 1500 per year	24 (36.9)	66 (78.6)	
Level of education, no. (%)			
Bachelor’s degree or above	39 (60.0)	21 (25.0)	< 0.001*
Other	26 (40.0)	63 (75.0)	
Confirmed diagnosis to surgery interval, no. (%)			
≤ 3 days	50 (76.9)	38 (45.2)	0.0001*
> 3 days	15 (23.1)	46 (54.8)	
Admission to surgery interval, no. (%)			
≤ 3 days	33 (50.8)	24 (28.6)	0.0068*
> 3 days	32 (49.2)	60 (71.4)	
Pathological type before surgery, no. (%)			
Invasive carcinoma	41 (63.1)	67 (79.8)	0.0644
Carcinoma in situ	9 (13.8)	8 (9.5)	
Others	15 (23.1)	9 (10.7)	
Confirmed diagnosis before surgery, no. (%)			
Yes	50 (76.9)	75 (89.3)	0.0465*
No	15 (23.1)	9 (10.7)	
Clinically positive nodes, no. (%)			
Yes	15 (23.1)	28 (33.3)	0.2036
No	50 (76.9)	56 (66.7)	
Tumor size (mm) (mean ± SD)	17.32 ± 6.31	18.10 ± 6.68	0.4696

*BCS* breast-conserving surgery

*P < 0.05

Interestingly, when the time interval between the diagnosis of breast cancer and surgery was > 3 days, the patient was significantly more likely to choose mastectomy than BCS (*P* = 0.0001). The same was the case when the interval from admission to surgery was more than 3 days (*P* = 0.0068). Patients with a confirmed diagnosis before surgery were also more likely to choose mastectomy than BCS (*P* = 0.046).

**Fig. 2:**
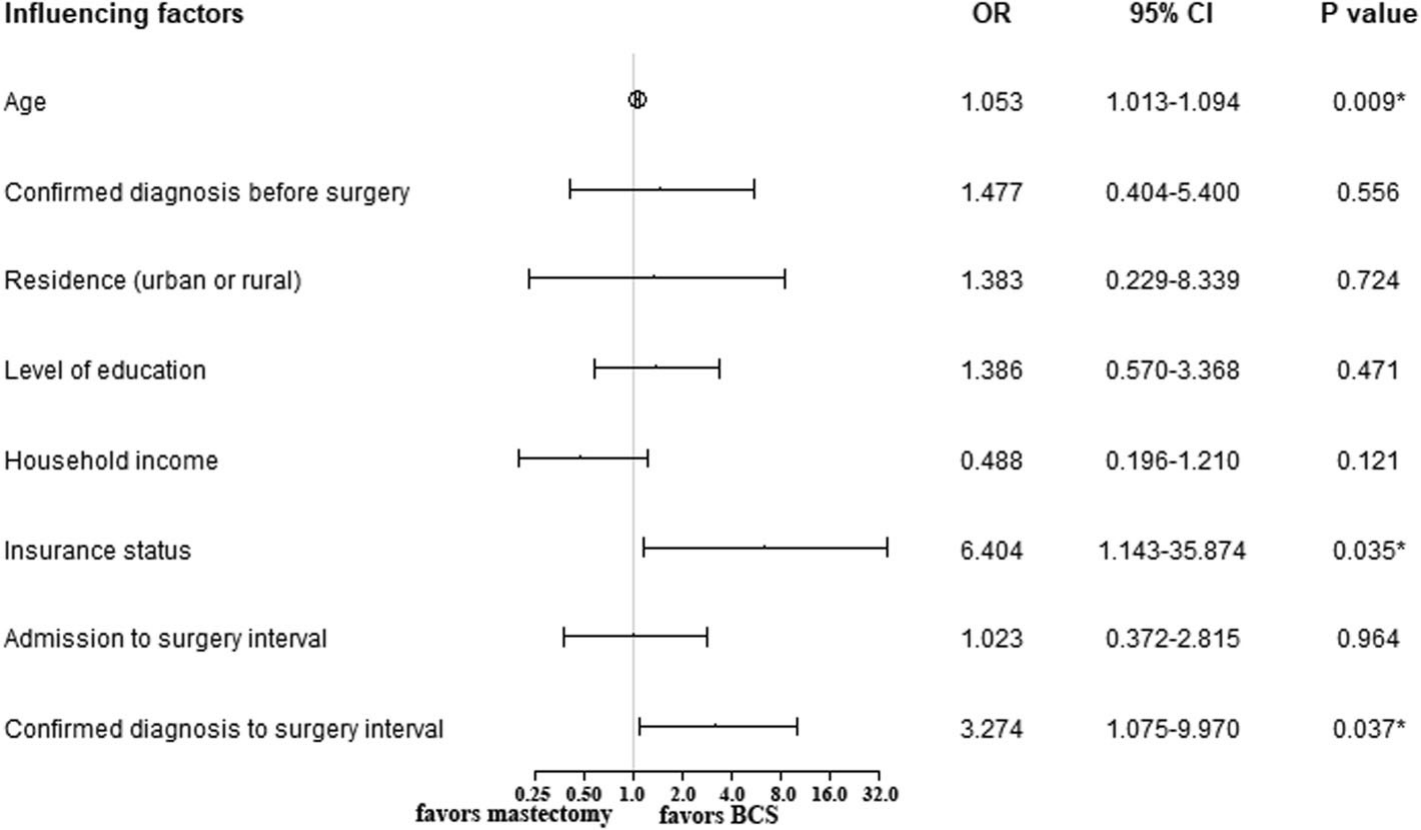
Multivariate analysis of the factors associated with the choice of BCS rather than mastectomy in patients who were eligible for BCS. OR, odds ratio; CI, confidence interval

Figure 2 summarizes the results of the multivariate analysis. Variables entered in the multivariate model included age at diagnosis, whether the diagnosis was confirmed before surgery, residence (urban or rural), level of education, household income, insurance status, admission to surgery interval, and confirmed diagnosis to surgery interval. This analysis revealed that the independent factors that influenced the choice between BCS and mastectomy were insurance status (general medicare insurance vs. no or low-level medicare insurance) (OR 6.404, 95% CI 1.143–35.874, *P* = 0.035), confirmed diagnosis to surgery interval (OR 3.274, 95% CI 1.075–9.970, *P* = 0.037), and age (OR 1.053, 95% CI 1.013–1.094, *P* = 0.009).

**Fig. 3:**
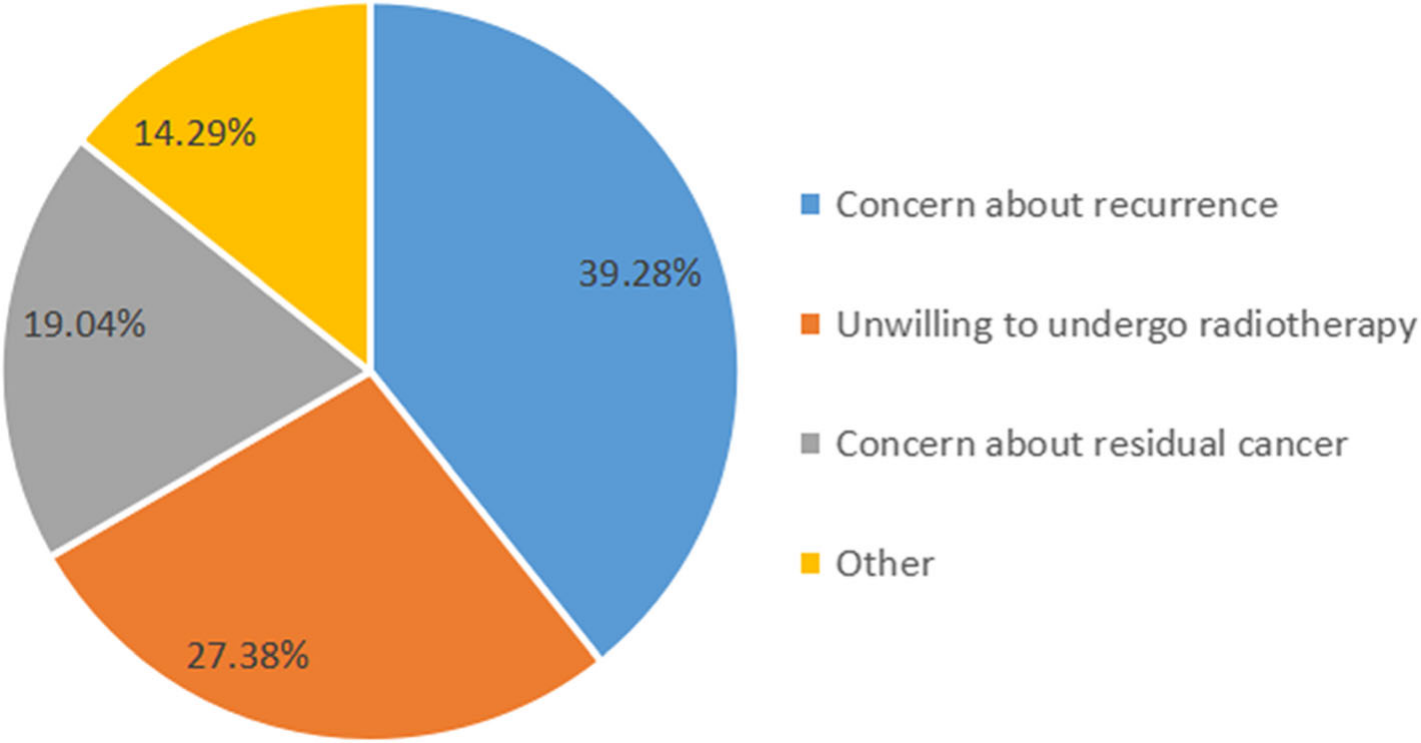
The questionnaire regarding the main reason for 84 patients choosing mastectomy rather than BCS among patients who were eligible for BCS

In the questionnaire about the main reason for choosing mastectomy rather than BCS, 33 (39%) of the 84 patients were concerned about recurrence of the cancer, 23 (27%) were unwilling to undergo radiotherapy, 16 (19%) were concerned about residual cancer, and 12 (14%) made the decision for other reasons (Fig. 3).

Table 2 summarizes the subanalysis of patients who had undergone NCT. A total of 46 patients received NCT. Among them, 16 were eligible for BCS after NCT, but only 2 patients chose BCS, with the remaining 14 choosing mastectomy with or without reconstruction. Among patients who did not receive NCT, 133 patients were eligible for BCS, of which 63 chose BCS and 70 chose mastectomy. Therefore, patients who received NCT were more likely to choose mastectomy compared with those who did not receive NCT (*P* = 0.0077). In the univariate analysis, there were no statistically significant differences in factors, including age, menstrual status, residence, insurance status, household income, and level of education for the two groups.

**Table 2:** Characteristics of the patients eligible for breast-conserving surgery who received or did not receive neoadjuvant chemotherapy (NCT) before surgery

Patient characteristics	NCT (*n* = 16)	No NCT (*n* = 133)	*P* value
Surgical choice, no. (%)			
BCS	2 (12.5)	63 (47.4)	0.0077*
Mastectomy	14 (87.5)	70 (52.6)	
Age, years			
Mean ± SD	48.69 ± 10.63	52.98 ± 12.91	0.2037
Median (range)	50.5 (31–64)	51 (25–85)	
Menstrual status, no. (%)			
Premenopausal	10 (62.5)	67 (50.4)	0.4329
Postmenopausal	6 (37.5)	66 (49.6)	
Place of residence, no. (%)			
Urban	10 (62.5)	104 (78.2)	0.2094
Rural	6 (37.5)	29 (21.8)	
Within Nanjing region	10 (62.5)	81 (60.9)	1.0
Outside Nanjing	6 (37.5)	52 (39.1)	
Insurance status, no. (%)			
General medicare insurance	10 (62.5)	95 (71.4)	0.5625
No or low-level medicare insurance	6 (37.5)	38 (28.6)	
Household income, $, no. (%)			
> 1500 per year	4 (25)	55 (41.4)	0.2820
≤ 1500 per year	12 (75)	78 (58.6)	
Level of education, no. (%)			
Bachelor’s degree or above	7 (43.8)	53 (39.8)	0.7920
Other	9 (56.2)	80 (60.2)	
Pathological type before surgery, no. (%)			
Invasive carcinoma	16 (100)	92 (69.2)	0.2137
Carcinoma in situ	0 (0)	17 (12.8)	
Others	0 (0)	24 (18.0)	
Confirmed diagnosis before surgery, no. (%)			
Yes	16 (100)	110 (82.7)	0.1339
No	0 (0)	23 (17.3)	
Tumor size (mm) (mean ± SD), no. (%)	17.18 ± 14.20	18.39 ± 6.01	0.5315

*P < 0.05

## Discussion

Not every patient with breast cancer is suitable for BCS. Contraindications are well defined and include large tumor size; cancer that is diffuse, multicentric, or multifocal; and a contraindication to radiation therapy [14, 15]. In addition, nipple discharge and tumors invading or close to the nipple are also considered as relative contraindications for BCS in many parts of China. China has special characteristics—such as doctor–patient relationship and medical expenses—that are different from those in developed countries; accordingly, Chinese practice is relatively conservative and cautious in the choice of various treatments, including surgical methods. In this study, we referred to the guidelines of CACA-CBCS, where the descriptions of contraindications for BCS were as follows: (1) absolute contraindications—have contraindications to radiotherapy; inflammatory breast cancer; wide lesions or confirmed as multicenter lesions, extensive or diffuse distribution of malignant microcalcifications, and difficult to achieve negative margin or ideal shape; the tumor has a positive margin following extensive local resection, and the pathological margin is still not guaranteed following re-excision, and (2) relative contraindications—poor tolerance to radiotherapy, tumor diameter > 5 cm, close to or invading the nipple (nipple Paget disease), imaging findings of multicenter lesions, and others. In this study, 122 patients were excluded because of these contraindications. Of the remaining 149 patients, 65 chose BCS and 84 chose mastectomy. The rate of BCS was 43.6% (65/149), indicating that numerous patients chose mastectomy despite being given the option of BCS.

Age was one of the factors shown in this study to significantly affect the choice of surgery [11, 12]. Older patients usually pay less attention to cosmetic outcomes, and they are more concerned about the impact of radiotherapy on their bodies. For these reasons, they are more willing to choose mastectomy. Teh et al. [16] found that patients in Asia older than 60 years were more willing to undergo mastectomy. The present study found that the patients who chose BCS were younger than those who chose mastectomy and that age at diagnosis was an independent factor influencing the patients’ choice of surgical method.

It has been suggested that patients of lower socioeconomic status (SES) may be less likely to choose mastectomy. In the present study, patients who resided in rural regions, those without general medicare insurance, those with a lower household income, and those with a lower level of education were all more likely to choose a mastectomy rather than BCS. Of these factors, the multivariate analysis showed that whether or not a patient had medicare insurance was an independent factor influencing their choice. In a review of 25 articles published in 7 databases between January 2000 and June 2014, Gu et al. [17] found that SES was associated with a higher rate of undergoing BCS. Kotwall et al. [18] also suggested that a low level of medicare insurance may be related to a patient choosing mastectomy. Hershman et al. [19] used the US 2000 census to generate an aggregate SES score for each zip code based on income, poverty, and education data; they found that patients who underwent mastectomy usually had lower SES. For patients with lower SES, the radiotherapy after BCS can be costly, particularly for those without general medicare insurance who pay the treatment costs themselves. In addition, the extra time and travel needed for radiation therapy after BCS may influence the treatment choice, and the complications associated with radiotherapy also have an influence. Patients with a lower level of education and outdated ideas may find it difficult to accept information based on new research. Some patients still hold the view that mastectomy is the only reliable choice for breast cancer surgery, believing a bigger operation would provide more effective treatment [20].

Core needle biopsy is now performed routinely for patients with suspected breast cancer to obtain a pathological diagnosis before surgery; because of this, many patients are diagnosed with breast cancer preoperatively. However, in some patients, the diagnosis was confirmed with intraoperative frozen pathological examination because a pathological diagnosis preoperatively for these patients was difficult to obtain. In the present study, patients with a confirmed diagnosis before surgery were more likely to choose mastectomy, as were those with a confirmed diagnosis to surgery interval of more than 3 days, which was shown to be an independent factor influencing the choice of a mastectomy. This factor has not been mentioned in previous studies. A possible reason why the patients with a confirmed diagnosis chose to have a mastectomy was that they were scared and panicked when informed they had breast cancer and wanted the lesion to be removed completely. Those with a longer interval between diagnosis and surgery may have been more susceptible to external factors, such as family, friends, and other patients.

The patients who chose mastectomy instead of BCS were asked before surgery to complete a questionnaire about the reason for their choice (Appendix). The main reasons were concern about recurrence of the cancer (39%) and unwillingness to undergo radiotherapy (27%). Previous studies have identified that patients feel safer after mastectomy and that the fear of recurrence is the primary motivator for choosing a mastectomy over BCS [20]. The fear of recurrence remains an issue in the long term, which is a challenge [21].

By downstaging the tumor, NCT can convert patients who would have undergone mastectomy into candidates for BCS. In addition, it can reduce excision volumes in patients with large tumors who are already candidates for BCS, thereby improving cosmetic outcomes. BCS after NCT has been shown to have an equivalent local-regional recurrence rate and overall survival rate when compared with no NCT before BCS [22, 23]. In the present study, 46 patients received NCT, of whom 16 were evaluated to be eligible for subsequent BCS. However, only two of these patients chose BCS, which was a significantly lower proportion than among the patients who did not receive NCT. This finding was similar to that of a large clinical study in China [24]. A possible explanation for this finding is that all the patients who underwent NCT were informed they had a relatively late-stage tumor; they may therefore have been less concerned about cosmetic outcome than about the risk to their lives. In addition, the long-term chemotherapy would have had an impact on their body and mind; they may have been too exhausted to accept the need for additional radiotherapy. It is likely that they may also have been influenced by external factors over time, particularly by other patients with breast cancer. There were few studies focusing on this issue.

It is important that further information regarding the surgical choice, such as prognosis, recurrence risk, complications, costs, and latest treatment concepts, should be provided to patients with breast cancer. Surgeons should help to overcome their psychological barriers and provide adequate explanation for their concerns and confusion. Patients should be more involved in their treatment process and choose the treatment approach that is best-suited for their situation because increased patient involvement is associated with increased patient satisfaction following the completion of treatment.

Several previous studies have investigated factors that influenced the surgical choice of patients with breast cancer [25, 26]. These were mostly retrospective and based in countries other than China. In particular, they did not eliminate interference from surgeons in the decisions, and it was frequently unclear whether patients were explicitly given the opportunity to make the surgical choice [13, 27]. The present study used a prospective design, and the patients eligible for BCS were given a genuine free choice between BCS and mastectomy after being given evidence that there was no difference in outcome between mastectomy and BCS and informed of the importance of radiotherapy as part of the treatment. However, this study was limited to a single institution and a fairly small sample size. We look forward to large clinical studies in China.

## Conclusions

Surgical choice of patients eligible for BCS was affected owing to several factors. Understanding the factors will allow surgeons and patients to engage in a fully informed preoperative decision-making process.

## Data Availability

Datasets from the current study are available from the corresponding author on reasonable request.

## Appendix

**Table 3:** Questionnaire for choosing mastectomy in patients who were eligible for BCS

Your age	( ) years
Your menstrual status	Premenopausal ( )
	Postmenopausal ( )
Your address	Urban ( ); rural ( )
	Within Nanjing ( ); outside Nanjing ( )
Your family income per year	> $1500 ( )
	≤ $1500 ( )
Your insurance status	General medicare insurance ( )
	No or low-level medicare insurance ( )
Level of education	Bachelor’s degree or above ( )
	Other ( )
Main reason for choosing mastectomy	Concern about cancer recurrence ( )
	Reluctance to undergo radiotherapy ( )
	Concern about residual cancer ( )
	Other reasons ( )

Please fill in your age and mark up “√” in the bracket after the option that you consider appropriate

## Abbreviations

BCS: Breast-conserving surgery
CACA-CBCS: Chinese Anti-Cancer Association, Committee of Breast Cancer Society
CI: Confidence interval
NCT: Neoadjuvant chemotherapy
OR: Odds ratio
SES: Socioeconomic status
